# Adaptation of acneic and non acneic strains of *Cutibacterium acnes* to sebum‐like environment

**DOI:** 10.1002/mbo3.841

**Published:** 2019-04-04

**Authors:** Valérie Borrel, Andrei V. Gannesen, Magalie Barreau, Charlotte Gaviard, Cécile Duclairoir‐Poc, Julie Hardouin, Yoan Konto‐Ghiorghi, Luc Lefeuvre, Marc G. J. Feuilloley

**Affiliations:** ^1^ Laboratory of Microbiology Signals and Microenvironment LMSM EA4312 University of Rouen Normandy, Normandie Université Evreux France; ^2^ Laboratory of Viability of Microorganisms of Winogradsky Institute of Microbiology, Federal Research Center “Fundamentals of Biotechnologies” Russian Academy of Sciences Moscow Russia; ^3^ R&D Uriage Dermatologic Laboratory Neuilly sur Seine France; ^4^ Laboratory « Polymères, Biopolymères, Surfaces » (UMR 6270 CNRS) Proteomic Platform PISSARO University of Rouen Mont‐Saint‐Aignan France

**Keywords:** bacterial adaptation, bacterial surface polarity, biofilm, cytotoxicity, inflammation, proteome analysis, sebum‐like medium

## Abstract

*Cutibacterium acnes*, former *Proprionibacterium acnes*, is a heterogeneous species including acneic bacteria such as the RT4 strain, and commensal bacteria such as the RT6 strain. These strains have been characterized by metagenomic analysis but their physiology was not investigated until now. Bacteria were grown in different media, brain heart infusion medium (BHI), reinforced clostridial medium (RCM), and in sebum like medium (SLM) specifically designed to reproduce the lipid rich environment of the sebaceous gland. Whereas the RT4 acneic strain showed maximal growth in SLM and lower growth in RCM and BHI, the RT6 non acneic strain was growing preferentially in RCM and marginally in SLM. These differences were correlated with the lipophilic surface of the RT4 strain and to the more polar surface of the RT6 strain. Both strains also showed marked differences in biofilm formation activity which was maximal for the RT4 strain in BHI and for the RT6 strain in SLM. However, cytotoxicity of both strains on HaCaT keratinocytes remained identical and limited. The RT4 acneic strain showed higher inflammatory potential than the RT6 non acneic strain, but the growth medium was without significant influence. Both bacteria were also capable to stimulate β‐defensine 2 secretion by keratinocytes but no influence of the bacterial growth conditions was observed. Comparative proteomics analysis was performed by nano LC‐MS/MS and revealed that whereas the RT4 strain only expressed triacylglycerol lipase, the principal *C. acnes* virulence factor, when it was grown in SLM, the RT6 strain expressed another virulence factor, the CAMP factor, exclusively when it was grown in BHI and RCM. This study demonstrates the key influence of growth conditions on virulence expression by *C. acnes*and suggest that acneic and non acneic strains are related to different environmental niches.

## 1 INTRODUCTION

*Cutibacterium acnes*, usually known as *Proprionibacterium acnes*, was recently renamed in order to distinguish this high GC content and human skin associated bacterium from other related species (McDowell, Barnard, Liu, Li, & Patrick, 2016; Sholz & Kilian, 2016). However, this species remains itself highly heterogeneous with six main phylotypes IA1, IA2, IB, IC, II, and III (Dagnelie, Khammari, Dréno, & Corvec, 2018; McDowell, 2017). *Cutibacterium acnes* genome is characterized by a large stable core (>80% of chromosomal DNA) whereas 7%–11% of coding sequences should be species or even strain specific (Brzuszkiewicz et al., 2011). Genomic island, resulting probably from horizontal transfer, are also encoded in the *C. acnes*genome and CRISPR sequence analysis suggests that subtypes IA and IB, which appears associated to the more severe forms of acne, evolved from subtypes II and III through successive deletion events (Bruggemann, Lomholt, & Kilian, 2012). In 2013, a study realized by Fitz–Gibbon et al. in the pilosebaceous units of acneic and non acneic patients revealed that, in skin, some ribotypes of *C. acnes* such as RT4 and RT5 should be associated to acne whereas RT6 clones should colonize non acneic skin. RT4 and RT5 strains are subtype IA and their genome contains a specific locus from plasmid origin (Locus 3) that should encode for virulence effectors (Fitz‐Gibbon et al., 2013).

Nevertheless, particularly in the case of *C. acnes*, associating genomic organization to virulence and pathophysiology should be done with the greatest cautions. Indeed, a proteomic analysis of the secretome of the four subtypes of *C. acnes* revealed a limited number of common proteins, except triacylglycerol lipase (GheA) considered as the principal virulence factor of this species, CAMP co‐hemolytic enzymes involved in pore formation, and glyceraldehyde 3‐phosphate dehydrogenase (GAPDH) required for bacterial adhesion (Holland et al., 2010). Moreover, for a same strain, expression of these proteins should depend on culture conditions as observed by comparing the proteomes of bacteria grown in brain heart infusion medium (BHI), egg yolk supplemented BHI, and reinforced clostridial medium (RCM) (Yu, Champera, & Kim, 2015). The major concern is that none of these media actually mimics the lipidic environment of *C. acnes* in the sebaceous gland which is its principal natural environment and where it can switch from a planktonic to a biofilm mode of development (Coenye, Honraet, Rossel, & Nelis, 2008). Because of its density, sebum is hardly compatible with experimental studies and different formulations of sebum like medium (SLM) have been proposed (Spittaels & Coenye, 2017; Stefaniak, Harvey, & Wertz, 2010; Wertz, 2009) and even patented (Catroux, Billoni, Cotovio, Buan, & Rubinstenn, 2005). Then, it was interesting to document the adaptation of distinct and well characterized strains *C. acnes* to different environments.

In this study, we compared the growth kinetics of RT4 acneic and RT6 non acneic strains of *C. acnes* in different culture media, including a specific SLM. The affinity of bacteria for the media was correlated with their surface polarity and biofilm formation activities. The influence of the environment on their intrinsic virulence, inflammatory potential, and proteome was also evaluated. This study indicates that acneic and non acneic strains have marked difference of affinity to polar and non polar environments suggesting that these strains should develop in specific ecological niches. Growth media have a specific impact on the expression of *C. acnes* virulence factors. Conversely, no correlation was found between the origin of bacteria, the polarity of the environment and their cytotoxic or inflammatory potential.

## 2 MATERIALS AND METHODS

### 2.1 *Cutibacterium* strains and culture conditions

The acneic strain ribotype 4 (RT4) HL045PA1/HM‐516 and the non acneic strain ribotype 6 (RT6) HL110PA3/HM‐554 of *C. acnes*(former *P. acnes*)*,*initially isolated by Fitz‐Gibbon et al. (2013), were diffused by BEI Resources American Type Culture Collection (Virginia, United States). These strains refer to phylotypes IA_1_ and II, respectively (McDowell, 2017). Bacteria stored at −80°C were initially plated on agar brain heart infusion (BHI, Becton Dickinson) as recommended by BEI resources. As these strains were almost unable to grow in aerobic conditions and on any medium tested, the plates were incubated under anoxic conditions at 37°C using BD GasPack^™^ or a Whitley A85 Workstation.

Colonies were transferred into sterile conical 15 ml tubes (Falcon) filled to maximal capacity with BHI or RCM and grown at 37°C. Bacteria were collected after 24 hr (exponential growth phase) or 72 hr (stationary phase) and sub‐cultured at 37°C in anoxic conditions in BHI, reinforced clostridial medium (RCM, Sigma Aldrich), or SLM. Growth monitoring was performed using a Xenius XMA microplate reader (SAFAS). Inoculum was added in 96‐well flat‐bottomed polystyrene plates (NUNC) to an initial OD_580_ = 0.08. Plates were prepared in anoxic conditions and sealed with Parafilm before incubation. Optical density of cultures was determined automatically every 15 min. Growth curves were determined over a minimum of three independent experiments.

### 2.2 SLM production

Considering that in *C. acnes* more than 20% of secreted and surface proteins can change under the influence of growth conditions (Yu et al., 2015) and the principal ecological niche of this bacterium in skin is the sebaceous gland, we decided to grown RT4 and RT6 strains in a SLM. Sebum is a dense medium incompatible to monitor bacterial growth. In addition, the principal components of sebum are highly hydrophobic molecules and they can easily form micelles and an unstable emulsion. Considering data from the literature and existing patents (Catroux et al., 2005; Stefaniak et al., 2010; Wertz, 2009) we decided to produce an RCM based medium supplemented with squalene, trioleine, oleic acid, and jojoba oil. The four lipidic compounds, initially dissolved individually in chloroform:methanol, 2:1 (100 mg/ml), were pooled in a glass tube and evaporated at 45°C under nitrogen (N_2_). After complete drying, RCM was added leading to a final medium containing squalene (6.3 mg/ml), trioleine (22.5 mg/ml), oleic acid (8,6 mg/ml), and jojoba oil (12.6 mg/ml). Tween 80 (15 g/L) was added to improve the solubility of these hydrophobic compounds. Upon addition of RCM, the medium was sonicated for 1 min in a bath sonicator and vortexed again over 1 min. SLM was homogeneous and remained stable for a minimum of 72 hr.

### 2.3 Characterization of *Cutibacterium*surface polarity

RT4 acneic and RT6 non acneic strains of *C. acnes* cultured in BHI or RCM were collected in stationary phase. The study was not realized using SLM as this technique requires quantities of medium not compatible with its preparation complexity and price. Bacteria were harvested (7,000×*g*; 10 min) and washed twice in sterile physiological water NaCl 0.9% (PS). The surface polarity and Lewis acid‐base balance of bacteria was studied using the microbial adhesion to solvents (MATS) technique (Bellon‐Fontaine, Rault, & Oss, 1996) and two solvent couples: chloroform/hexadecane and ethyl acetate/n‐decane. For each bacterial strain and growth condition, 2.6 ml of bacterial suspension at OD_400_ 0.8 was mixed for 60 s with 0.4 ml of each solvent indicated above. The OD of the aqueous phase was measured at 400 nm. The percentage of cells in each solvent was calculated using the following equation: (1 − A/A_0_) × 100. The experiments were carried out in at least five replicates.

### 2.4 Measure of biofilm formation activity by CV staining

Biofilms were formed in 96‐well flat‐bottomed microtitration polystyrene plates (NUNC). Experiments were carried out according to the modified classical procedure (O'Toole, 2011). Bacteria were harvested (7,000×*g*; 10 min) and rinsed with PS. Hundred microliters aliquots of bacterial culture adjusted to OD_580_ = 1 were layered in microtitration plates and incubated for 2 hr to allow primary adhesion. Then, wells were washed with PS, media were added and plates were incubated for 72 hr in static and anoxic conditions. At the end of the study, wells were washed four times with PS to remove remaining planktonic bacteria. Biofilms were fixed with methanol for 15 min. After fixation, methanol was removed and the plates were dried and stained with 0.1% crystal violet (CV) for 10 min. After rinsing with PS, the dye was recovered by 100 µl of a combination of acetone:ethanol, (20:80, v/v) and the OD595 nm of the solution was measured on a Biorad spectrophotometer. Ten wells were used for every sample and experiments were carried out in at least three replicates.

### 2.5 Cytotoxicity studies

The cytotoxic potential of acneic and non acneic strains of *C. acnes* grown in BHI, RCM, or SLM was determined by the measurement of lactate dehydrogenase (LDH) release by HaCaT keratinocytes due to cytoplasmic membrane destabilization. HaCaT cells (CLS, Eppelheim, Germany) were cultured at 37°C in 5% CO_2_ atmosphere in Dulbecco's modified Eagle's medium (DMEM, Lonza) containing 25 mM glucose and supplemented with 10% inactivated fetal bovine serum, 2 mM L‐glutamine (Lonza), and antibiotics (penicillin 100 IU/mL and streptomycin 100 μg/mL). Cells were used between passages 41 and 65. They were seeded in 24 well plates at a final density of 5 × 10^5^ cells per well, and grown for 48 hr before use. A minimum of 8 hr before interaction with bacteria, cells were starved of antibiotics and fresh serum‐free medium was added. Bacteria were harvested (7,000×*g*; 10 min), washed in PS and HaCaT cells were infected at a bacterium‐to‐cell ratio of 50:1. The amount of LDH released by HaCaT cells was determined after 18 hr of incubation using the Cytotox 96 enzymatic assay (Promega, France) (Picot et al., 2003). Control studies performed using bacteria alone showed that none of the strains used in this study produced metabolites interfering with the assays.

### 2.6 IL8, interleukin 1α, and human defensing‐2 secretion studies

The inflammatory response of HaCaT cells to acneic and non acneic strains of *C. acnes* grown in BHI, RCM, or SLM was evaluated by assaying interleukin 8 (IL8) and interleukin 1α (IL1α) secretion in the culture medium. HaCaT cells were exposed to bacteria as previously described (N'Diaye et al., 2016). The amount of IL8 and IL1α released by HaCaT cells was determined after 18h of incubation using Human IL‐8 ELISA Kit (KHC0081) (Invitrogen, ThermoFisher scientific) and Human IL‐1α/IL‐1F1 Quantikine ELISA (cat. DLA50) (R&D SYSTEMS, bio‐techne brand) respectively. Human defensin‐2 (HBD2) secretion was assayed using a Human β‐Defensins 2 ELISA kit (cat. CSB‐E13201h) according to the manufacturer's protocol (Cusabio, Wuhan Hi‐tech Medical Devices Park, China).

### 2.7 Total proteome analysis

Acneic and non acneic strains of *C. acnes* cultured in BHI, RCM, or SLM were collected in early stationary phase by centrifugation (10,000×*g*for 10 min) and rinsed twice with Tris‐HCl pH 7,4 buffer (20 mM) supplemented with protease inhibitors (Boehringer, Paris, France). Three freeze/thawing cycles were applied to the suspension (−80°C for 10 min/37°C for 5 min). Bacterial lysis was completed by sonication using a BRANSON Digital Sonifier (Danbury, Connecticut) equipped with a 250 W horn. Samples (kept in melting ice to avoid temperature increase) were submitted to 12 sonication cycles (Program 4, 30 s sonication—30 s latency). Bacterial lysis was controlled by microscopic observation. Cell lysates were subsequently centrifuged at 13,000×*g* for 10 min at 4°C. Supernatants were collected and treated with RNAse and DNAse and then analyzed by tandem mass spectrometry using a LTQ‐Orbitrap Elite coupled with an Easy nLC II system (Thermo Scientific, Villebon‐sur‐Yvette, France). The samples were injected onto an enrichment column (C18 PepMap100, Thermo Scientific, Villebon‐sur‐Yvette, France). Separation was realized with an analytical column needle (NTCC‐360/100‐5‐153, NikkyoTechnos, Tokyo, Japan). The mobile phase consisted of H2O/0.1% FA (buffer A) and ACN/FA 0.1% (buffer B). Tryptic peptides were eluted at a flowrate of 300 nl/min using a three‐step linear gradient: from 2% to 40% B over 25 min. The mass spectrometer was operated in positive ion mode with a capillary voltage and a source temperature set at 1.5 kV and 275°C, respectively. The samples were analyzed using the collision induced dissociation method. The first scan (MS spectra) was recorded in the Orbitrap analyzer (*R* = 60,000) in the mass range m/z 400–1800. The 20 most intense ions were then selected for MS2 experiments. Singly charged species were excluded for MS2 analysis. Dynamic exclusion of already fragmented precursor ions was applied for 30 s, with a repeat count of 1, a repeat duration of 30 s, and an exclusion mass width of ±10 ppm. Fragmentation occurred in the linear ion trap analyzer with collision energy of 35. All measurements in the Orbitrap analyzer were performed with on‐the‐fly internal recalibration (lock mass) at m/z 445.12002 (polydimethylcyclosiloxane). Raw data files were processed using Proteome Discoverer 1.3 software (Thermo Scientific, Villebon‐sur‐Yvette, France). Peak lists were searched using the MASCOT search engine (Matrix Science, Boston, Massachusetts) against the database Swiss Prot database. Searches were performed with the following parameters: 1 missed trypsin cleavage site allowed; variable modifications: carbamidomethylation of cystein, and oxidation of methionine; mass tolerance on parent and daughter ions: 10 ppm and 0.5 Da, respectively. Only proteins represented by more than one peptide sequence, by a minimum of a twofold variation and found in the three repeats in each medium were retained. Each protein identification was validated by an ANOVA test. All identified proteins were classified in regard to their function using UniProt (https://www.uniprot.org) and EMBL‐EBI InterPro (https://www.ebi.ac.uk/interpro).

### 2.8 Statistical treatment of the data

The data were analyzed using the Mann–Whitney non parametric criterion. Graph plotting and calculation of the Mann–Witney criterion were carried out using Microsoft Excel 2007 (R software). The differences between experimental and control variants were considered reliable at confidence coefficient >95%.

## 3 RESULTS

### 3.1 Acneic and non acneic strains of *C. acnes* are adapted to different environments

**Figure 1:**
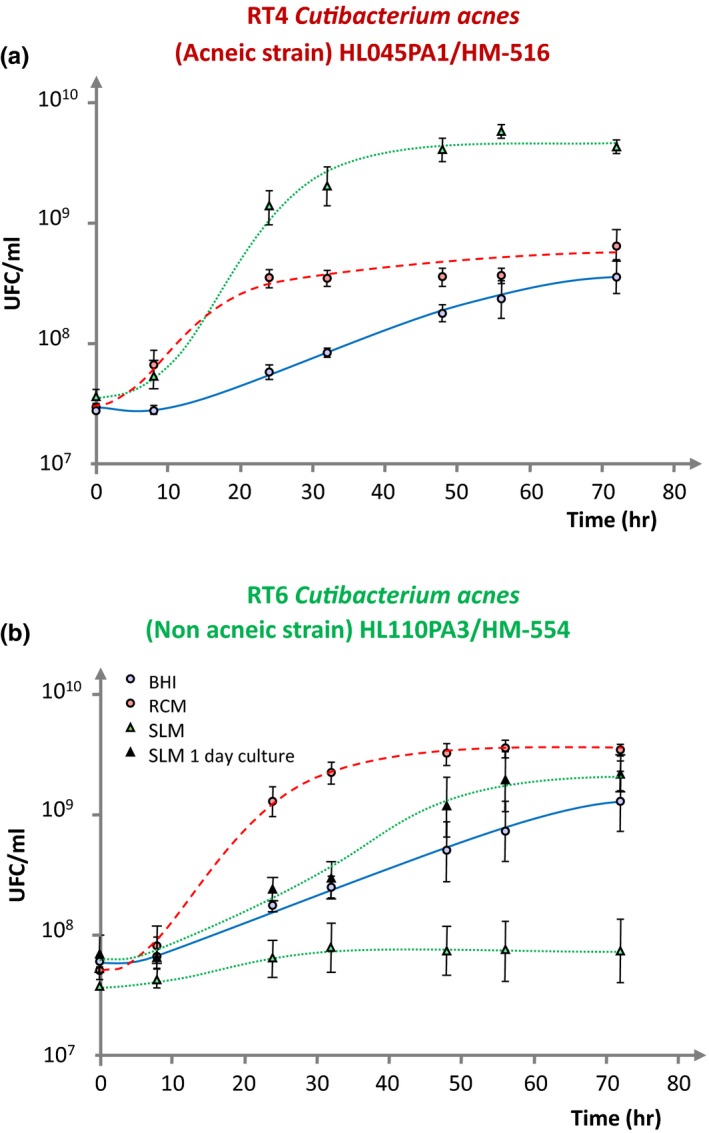
Comparative growth kinetics of RT4 acneic (a) and RT6 non acneic (b) strains of *Cutibacterium acnes* in brain‐heart infusion (BHI) , reinforced clostridial medium (RCM) and sebum‐like medium (SLM) after 3 days and 1 day preculture in RCM. Results are the mean ± SEM of a minimum of three independent experiments.

When the RT4 acneic and RT6 non acneic strains of *C. acnes* where grown in brain‐heart infusion (BHI), RCM, and sebum‐like medium (SLM) their behavior was markedly different. The RT4 acneic strain showed a maximal biomass formation and shorter generation time in SLM (Figure 1a). In RCM, its generation time was equivalent but the stationary growth phase was reached more rapidly and the total biomass formed was decreased (1 Log unit). The acneic strain was hardly growing in BHI, although after 70 hr of culture it reached a final biomass close to that obtained in RCM after 20 hr. The behavior of the RT6 non acneic strain was at the opposite. The highest biomass formation and shorter generation time were noted when this bacterium was grown in RCM (Figure 1b). Conversely these strains were almost unable to multiply in SLM. Growth was observed in BHI, although it remained much slower than in RCM. It should be noticed that all these studies were realized using bacteria obtained from 3 days old precultures, that is, in late stationary growth phase. When the same experiment was realized using 1‐day‐old precultures, that is, bacteria in earlier development phase, in SLM the RT6 non acneic strain showed a much more rapid growth kinetic and an important biomass formation which after 70 hr was close to that obtained in RCM.

### 3.2 The polarity and Lewis acid/base surface properties of acneic and non acneic strains of *C. acnes* are modified by the growth medium

**Figure 2:**
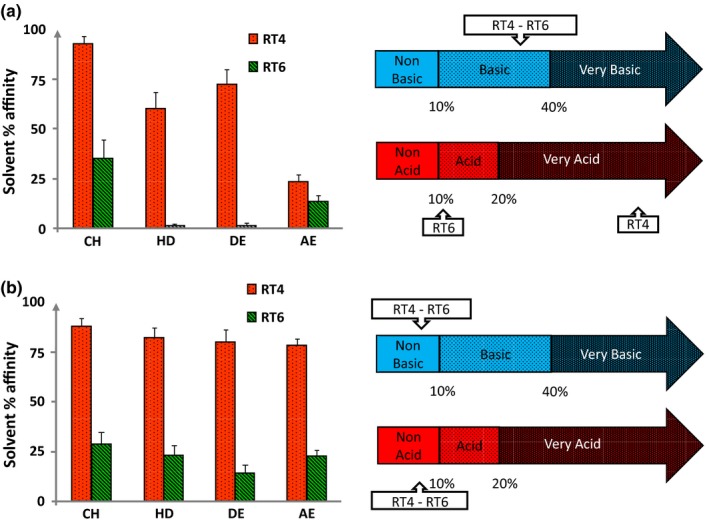
Affinity to solvents and Lewis acid/base surface properties of ribotype 4 (RT4) acneic and ribotype 6 (RT6) non acneic (RT6) strains of *Cutibacterium acnes* grown in (a) brain‐heart infusion medium (BHI) or (b) reinforced clostridial medium (RCM). Partition between water and solvents of different polarities (left) and Lewis acid‐base values (right) of RT4 and RT6 strains of *C. acnes*was studied by the MATS technique using chloroform (CH), hexadecane (HD), Decane (DE), and ethyl acetate (EA). Results are representative of five independent experiments

As the two strains of *C. acnes* showed totally different growth kinetics in normal and lipid rich growth media, we postulated that their surface polarity should be modified. This was investigated by the microbial affinity to solvents (MATS) technique. Using bacteria grown in BHI, we observed that the RT4 acneic strain showed a high affinity to solvents, particularly to apolar ones such as decane and hexadecane, whereas the affinity to ethyl acetate, a less hydrophobic solvent, was more limited (Figure 2a). The affinity of RT4 to chloroform can be explained by the Lewis basic character of its surface, as also indicated by its high affinity to decane. The surface properties of the RT6 non acneic strain grown in BHI were markedly different, with a low affinity to chloroform and ethyl acetate and a minimal affinity to hexadecane and decane. As a consequence, it appears that the RT6 strain grown in BHI was presenting a polar (low hydrophobicity) and intermediate Lewis basic/acid surface. Growing the RT4 acneic strain in RCM was leading to a reinforcement of its hydrophobic surface character with an affinity to the four solvents >75% (Figure 2b). As indicated by the absence of affinity to a specific solvent, these bacteria did not show basic or acid Lewis character. As in BHI, the RT6 strain grown in RCM showed a low hydrophobicity surface although its affinity to hexadecane and decane was increased. The major evolution of RT6 strain in RCM was the disappearance of its surface Lewis basic/acid character.

### 3.3 The biofilm formation activities of acneic and non acneic strains of *C. acnes* show opposite evolutions in regard to the medium polarity

**Figure 3:**
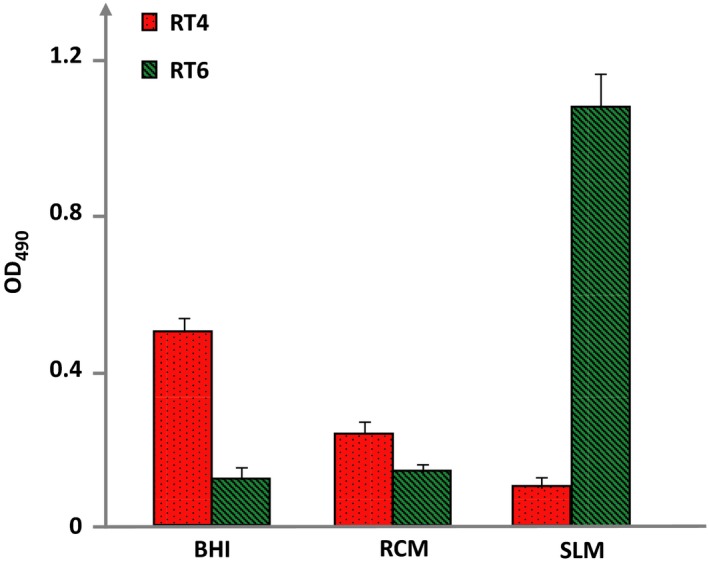
Biofilm formation activity of RT4 acneic and RT6 non acneic strains of *Cutibacterium acnes* grown in brain‐heart infusion (BHI), reinforced clostridial medium (RCM), and sebum‐like medium (SLM). Biofilm formation was measured by the crystal violet technique. Results are representative of three independent experiments

The biofilm formation activity of the RT4 acneic strain was favored when it was grown in the more polar medium, namely BHI (Figure 3). It was decreased when the bacterium was exposed to RCM and very low when it was produced in SLM. The behavior of the non acneic strain RT6 was the opposite with a low production of biofilm in BHI, higher in RCM, and maximal in SLM. There was consequently a complete antinomy between the surface polarity of the bacteria and their biofilm formation activity in the different media. Indeed, we observed a higher biofilm formation activity of the RT4 hydrophobic acneic strain in the more hydrophilic medium (BHI) and conversely a higher biofilm formation activity of the RT6 non acneic strain in the more hydrophobic medium (SLM).

### 3.4 Cytotoxicity, inflammatory, and β‐defensin 2 inducing potential of acneic and non acneic strains of *C. acnes* are poorly affected by growth conditions

**Figure 4:**
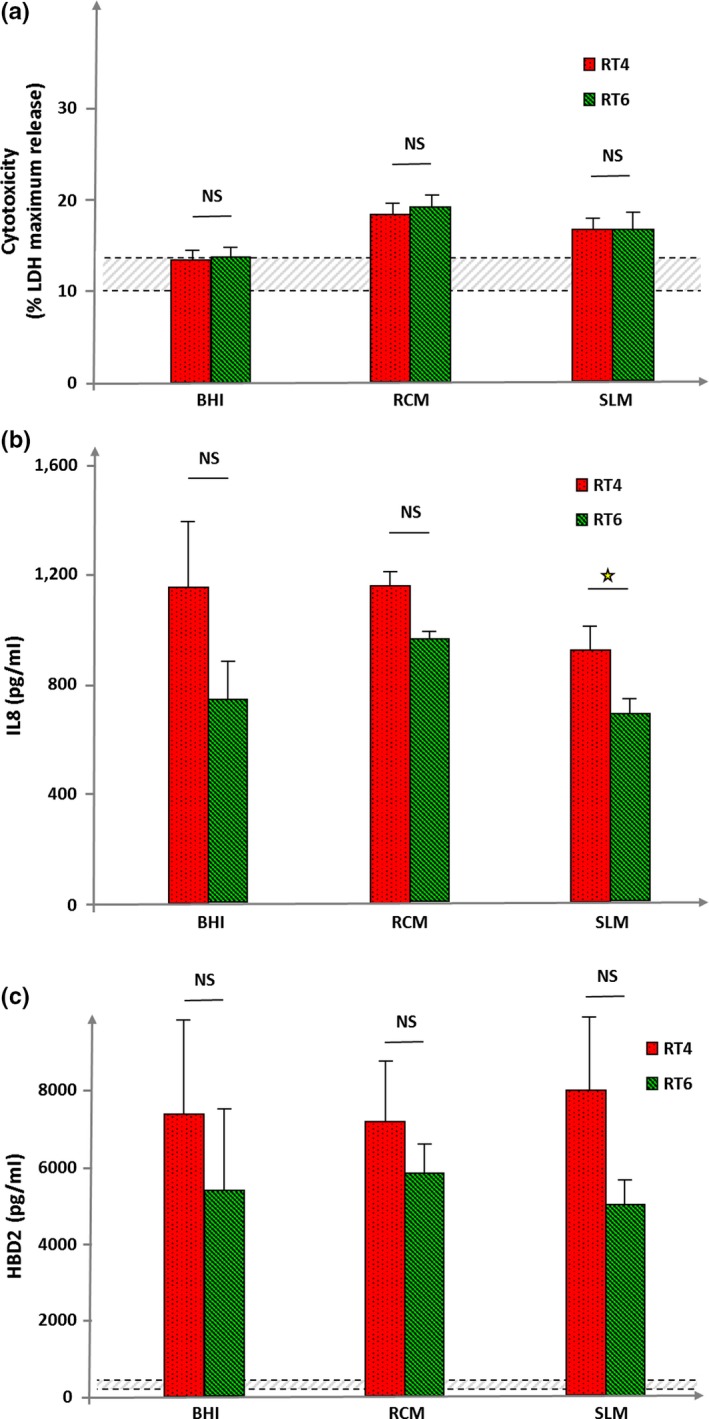
Cytotoxicity, inflammatory, and β‐defensin 2 inducing potential of RT4 acneic and RT6 non acneic strains of *Cutibacterium acnes*grown in brain‐heart infusion (BHI), reinforced clostridial medium (RCM), and sebum‐like medium (SLM). Cytotoxicity (a) was measured by assay of lactate dehydrogenase (LDH) released by HaCaT keratinocytes after exposure to bacteria. Cytotoxicity is expressed as percentage of maximal LDH release obtained by total lysis of cultured keratinocytes using Triton X100. Dotted lines indicate the mean spontaneous cell death percentage in control cultures. The inflammatory response of HaCaT keratinocytes after exposure to bacteria (b) was measured by assay of interleukin 8 (IL8) released in the culture medium. IL8 secretion was not detectable in control conditions. Human β‐defensin 2 (HBD2) secretion by HaCaT keratinocytes was measured in control cultures (bottom dotted line) and lysed cells (top dotted line) and after exposure to bacteria grown in the different media. Results are the mean ± SEM of a experiments (NS: not significantly different; ⋆ = *p* < 0.05)

The cytotoxicity of the two strains was studied on HaCaT keratinocytes by assay of LDH released using bacteria at an MOI = 50. Even in these conditions of high infection level, the two *C. acnes* strains showed limited cytotoxicity (Figure 4a). Bacteria were tested at higher concentrations (MOI = 100 and 200) but no increase in cytotoxicity was observed (*Data not shown*). Moreover, in all tested conditions, no difference of cytotoxicity was observed between acneic and non acneic bacteria. When bacteria were grown in BHI their cytotoxicity was close to the basal values of HaCaT spontaneous cell death. Bacteria grown in RCM showed a higher cytotoxicity, nevertheless, this level remained low, with a mean cell death between 19% and 23%. Bacteria grown in SLM showed a cytotoxicity of the same order as observed in RCM. Consequently, it appears that there was no correlation between the clinical character of the bacterium (acneic or not), its affinity for polar or apolar media, and its cytotoxic activity.

Considering that acne is more associated to inflammation than to cell death, we decided to test the effect of the two strains on IL8 as a marker of inflammation (Figure 4b). In the absence of bacteria, the basal production of IL8 by HaCaT keratinocytes was undetectable. Exposure to acneic or non acneic *C. acnes* was clearly associated to an increase in IL8 secretion. However, the production of IL8 induced by bacteria was not modified according to the strain or its growth conditions suggesting that the culture medium has no influence on the bacterial inflammatory potential. Nevertheless, in all cases, the RT4 acneic strain showed a higher IL8 inducing potential than the RT6 non acneic strain. This difference was particularly significant when the bacteria were grown in SLM. Interleukin 1α production was also assayed as a marker of early inflammatory response but the detectable quantity was very low and no variation was observed between the strains and growth conditions (*Data not shown*).

As keratinocytes generally react to bacteria by releasing antimicrobial peptides, we also investigated the effect of RT4 and RT6 *C. acnes*on β‐defensin 2 (HBD2) secretion by HaCaT cells (Figure 4c). In the absence of bacterial stimulation, the amount of HBD2 produced by HaCaT cells was minimal (between 135 and 220 pg/ml). Exposure to acneic or non acneic *C. acnes* resulted into a marked increase in HBD2. The effects of the non acneic strain were systematically lower than that of the acneic strain, but the differences were not significant. Moreover, there was no visible influence of the growth medium.

### 3.5 Acneic and non acneic strains of *C. acnes* express growth medium dependent proteins

**Figure 5:**
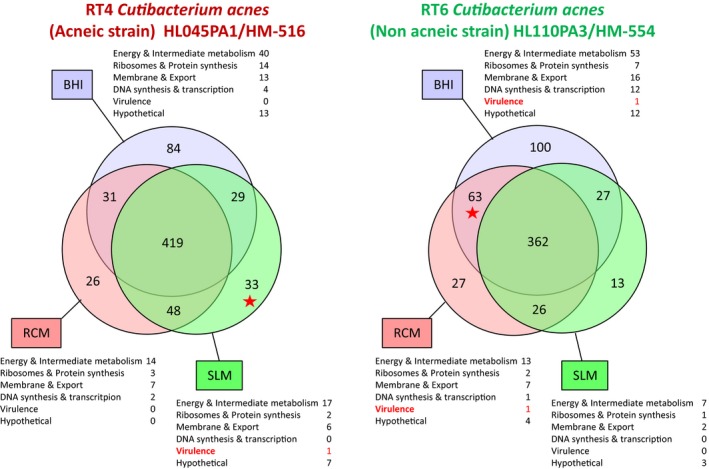
Venn diagrams illustrating the number of common and specific proteins expressed by RT4 acneic and RT6 non acneic strains of *Cutibacterium acnes* grown in brain‐heart infusion (BHI), reinforced clostridial medium (RCM), and sebum‐like medium (SLM). Proteins were classified according to their potential functions. The complete list of proteins expressed in each medium and specifically found only in BHI, RCM, or SLM by RT4 and RT6 strains of *C. acnes* is provided as Data S1 and Data S2

In order to go further into the metabolic influences of growth conditions on RT4 and RT6 *C. acnes* strains, a proteomic analysis was realized by nano LC‐MS/MS on total proteins expressed by bacteria in BHI, RCM, and SLM. Each strain showed a central core of common proteins and a limited number of specific for each growth medium (Figure 5). For the RT4 acneic strain, the core included a mean of 419 proteins whereas 84 were specifically expressed in BHI, 26 in RCM, and 33 in SLM. These proteins included essentially intermediate metabolism enzymes but also membrane and export proteins and, in a lower extend, factors involved in DNA and protein synthesis or regulation. We were particularly interested to visualize the presence of triacylglycerol lipase when the RT4 acneic strain was grown in SLM and not in BHI or RCM. For the RT6 non acneic strain, the common core included a mean of 362 proteins whereas 100 were specific of BHI, 27 of RCM, and 13 of SLM. These proteins were shared in the same categories as in the RT4 strain. We noted that the RT6 strain overexpressed a CAMP factor, typically associated to pore formation and virulence expression, when it was grown in BHI and RCM. Conversely, this factor was not detected in the proteome of SLM bacteria. The RT6 strain did not express triacylglycerol lipase in any of the tested growth conditions. GAPDH, a factor typically involved in *C. acnes* adhesion and virulence was not never found in the proteome of the RT4 and RT6 strains. The complete list of the molecules identified in the proteome of *C. acnes*(RT4) HL045PA1/HM‐516, and (RT6) HL110PA3/HM‐554 is presented in Data S1 and Data S2.

## 4 DISCUSSION

*Cutibacterium acnes* is an important member of the human skin microbiota (Christensen & Brüggemann, 2014). Although the taxonomy of this species was recently revised (Scholz & Kilian, 2016), *C. acnes*remains heterogeneous and has been sub‐divided in six principal phylotypes (Dagnelie et al., 2018). In skin, bacteria such as the RT4 HL045PA1/HM‐516 (phylotype IA_1_) appear predominantly associated to acne, whereas strains such as the RT6 HL110PA3/HM‐554 (Phylotype II) are found on healthy skin areas (Fitz‐Gibbon et al., 2013). BHI, classically proposed by strain libraries for *C. acnes* culture, showed mediocre performances although both strains were capable to use it. However, as observed herein, these two strains are presenting important differences of affinity in regard to culture media. Whereas the optimal growth medium of the RT4 acneic strain was SLM, that is, a medium supplemented with lipids aimed at representing sebum, the RT6 non acneic strain showed maximal growth in RCM, a protein rich medium but containing minimal amounts of lipids. Moreover, the RT4 strain was capable to grow in RCM, whereas the RT6 strain was almost unable to use SLM, at least when the inoculum was prepared from a 3 days old preculture. Indeed, when the inoculum was made from a 1‐day‐old preculture, that is, with bacteria in exponential growth phase, the RT6 strain was capable to grow in SLM and even better than in BHI. This suggests that bacteria in exponential growth phase, which show a higher metabolic activity, have a better adaptability than bacteria in stationary phase. These results suggest that acneic and non acneic strains are adapted to very different ecological niches although these results could also reflect more strain related differences.

These observations suggest that acneic and non acneic strains are adapted to different lipidic environments, we realized MATS studies in order to evaluate the surface polarity of these bacteria. In BHI, the RT4 strain showed a high affinity to apolar solvents and this characteristic was reinforced when it was grown in RCM. This is indicating that the surface of this strain is highly hydrophobic. In addition, whereas it was showing a very acid character and then surface charges when it was grown in BHI, in RCM it was neither basic nor acid, suggesting a total absence of ionized residues. Surface acidic polysaccharides (Nagaoka et al., 1985), peptidoglycans (Kamisango, Fujii, Yanagihara, Mifuchi, & Azuma, 1983), lipoglycans (Whale, Sutcliffe, Morrisson, Pretswell, & Emmison, 2004), and proteins (Yu et al., 2015) have been identified in *C. acnes* but its cell wall lipidome remains poorly documented. This study suggests that apolar residues should represent the essential of the surface in acneic *C. acnes*strains and this character should be regulated by the microenvironment. The very hydrophobic *C. acnes* RT4 surface can even interrogate on the capacity of this bacterium to scavenge lipids from its environment as observed in many bacterial species (Fozo & Rucks, 2016). On the contrary, the non acneic *C. acnes* RT6 strain showed limited affinity to solvents, and particularly when it was grown in BHI, with an almost absence of affinity for the more apolar solvents. Consequently, it appears that the RT6 strain cell wall should include essentially polar molecules. However, as observed with the RT4 strain, when the *C. acnes* RT6 strain was grown in RCM, it loosed its basic/acid character suggesting the absence of ionized residues.

This difference of affinity for lipidic or non lipidic environments and surface polarity of acneic and non acneic strains is coherent with results of biofilm formation activity. Indeed, when the RT4 strain was grown in the more polar medium (BHI) it was forming biofilm in abundance whereas this activity was reduced in RCM and even more in SLM. Biofilm is a defence mechanism for bacteria and a strategy to resist in unfavorable environments (Zhurina, Gannesen, Zdorovenko, & Plakunov, 2014), as for the RT4 in BHI. The same was observed, but at the opposite, with the RT6 strain. Indeed, the RT6 strain was forming a minimum of biofilm in BHI and RCM, but a large amount when it was grown in a lipid rich medium such as SLM, that is, a medium poorly adapted to its polar character. Then, in *C. acnes,* the biofilm formation activity appears clearly related to its surface properties and conditioned by its environment. The mechanisms underlying this balance between biofilm or planktonic development remain to be investigated in *C. acnes*, particularly in regard to a possible role of c‐di‐GMP and cAMP, as demonstrated in other bacterial species (Bouffartigues et al., 2015).

Unexpected results were obtained when cytotoxicity studies were realized. Indeed, acneic and non acneic *C. acnes* strains showed an equal cytotoxicity whatever was the growth medium employed. Moreover, their cytotoxic activity remained very low, close to the basal values when the bacteria were grown in BHI. A marginal increase was observed when the bacteria were grown in RCM or SLM. Then, the cytotoxicity, or presently the absence of cytotoxicity of acneic and non acneic *C. acnes* appears independent of their surface properties and growth conditions. However, *C. acnes* is not considered as a real pathogen and its role in acne is generally attributed to its triglyceride lipase activity leading to the production of acidic metabolites and inflammation (Holland et al., 2010). Then, IL8 was used as a marker of inflammation in HaCaT keratinocytes to investigate the potential differences between acneic and non acneic *C. acnes* grown in polar or apolar media. IL8 assays showed that the acneic RT4 strain was presenting a higher inflammatory activity than the RT6 strain. However, this effect was not modified following the bacterial growth medium and thus the lipidic environment. Interleukin 1α assays revealed that the production of this molecule by HaCaT cells in our experimental conditions was very low and no increase due to the bacteria was noted. Other cytokines were not assayed but it should be interesting to perform a more complete microarray study. Then, considering the present results the inflammatory potential of *C. acnes* appears as an intrinsic property, unrelated to its surface polarity. As antimicrobial peptides can be also stimulated by commensal or non commensal bacteria, acneic and non acneic strains of *C. acnes* were studied in regard to their capacity to stimulate HBD2 secretion by keratinocytes. The behavior of the two bacteria was consistent with our previous results on cytotoxicity and no influence of the culture conditions was observed. However, it remains interesting to note that the acneic RT4 strain systematically induced a higher production of HBD2 than the non acneic RT6 strain, although the difference was not significant.

The proteomic analysis of bacteria grown in the different media provides some explanations on their behavior in regard to eukaryotic cells. The acneic RT4 strain showed a central core of proteins (74%–80% of the total) expressed independently of the culture conditions. These proteins are involved in energy and intermediate metabolism, ribosomes and protein synthesis, membrane structure, and export as well as DNA synthesis and transcription. However, no molecule classically involved in *C. acnes* virulence was found in this central core. Expression of triacylglycerol lipase, one of the principal virulence factor of *C. acnes* (Holland et al., 2010), was only detected in the RT4 strain proteome when it was grown in SLM. The absence of known virulence factors in the proteome of bacteria grown in BHI or RCM is in agreement with their very limited virulence. Detection of triacylglycerol lipase exclusively by bacteria grown in SLM confirms the tropism of this strain for this lipid rich medium and suggests that in vivo, these bacteria should require the environment of sebaceous glands to express their virulence factors. However, the absence of cytotoxicity of this strain should appear incoherent with the expression of this major virulence factor. In fact, this should be explained by the requirement of host molecules, such as acid sphingomyelinase, for *C. acnes* virulence expression as shown by Nakatsuji, Tang, Zhang, Gallo, and Huang (2011), and it is not obvious that in our experimental conditions HaCaT keratinocytes express this enzyme in sufficient amount. In addition, other *C. acnes* virulence factors were not detected in the proteome of this strain. The non acneic RT6 strain also showed a central core of proteins expressed in the three tested media. It varied between 65% and 84% of the total in BHI and SLM, respectively, and appears more influenced by cultures conditions than for the RT4 strain. Proteins dispatched in the same categories as for the RT4 acneic strain. The unique virulence factors detected, in this strain was the CAMP co‐hemolytic enzyme, and it was found only in the proteome of bacteria grown in BHI and RCM. No known *C. acnes* virulence factor was found in the proteome of the RT6 strain grown in SLM. These results are in agreement with the data presented in Figure 1 indicating that the non acneic strain RT6 is not adapted to a lipidic environment. Moreover, they suggests that the RT4 and RT6 strains refer to different ecological niches although they were presented as collected from the same environment (nose pilosebaceous units, Fitz‐Gibbon et al., 2013). In fact, it appears that for a better understanding of bacteria interaction with skin, it should be necessary to be more precise in term of localization when bacteria are collected. On the basis of its physiological requirements, the non acneic RT6 strain, which shows relative tolerance to oxygen, should naturally inhabit the surface of the stratum corneum and upper part of the hair follicle. Conversely, the RT4 acneic strain, which behaves as an anerobic bacterium, is perfectly adapted to grow in the sebaceous glands. This tropism, as well as its higher relative inflammation potential is perfectly in coherence with an involvement in acne etiology.

Taken together the present data demonstrate that the in vitro study of the acneic potential of *C. acnes* requires the use of specific media which are necessary for the bacterium to express its pathogenic activity. This is also revealing that it should be essential to look more in details at the precise localization of bacterial strains within the different region of the skin and its appendages.

## CONFLICT OF INTERESTS

The authors have the following interests: This work was supported by public funds obtained from Evreux Porte de Normandie, Region Normandie, and European Union (FEDER). Luc Lefeuvre is employed by the Dermatologic Laboratories Uriage. There are no patents, products in development of marketed products to declare. This does not alter the authors’ adherence to all policies on sharing data and materials, as detailed online in the guide for authors.

## AUTHOR CONTRIBUTIONS

VB, AG, MR, JH, and CD‐P performed the experiments, analyzed the data and wrote the draft of the manuscript. YK‐G and LL supervised the work. MGJF headed funding organization, supervised the work, and assisted for manuscript writing. All authors read and approved the final manuscript.

## ETHICS STATEMENT

None required.

## Data Availability

All data are provided in full in the results section of this paper. The complete list of proteins expressed by bacteria in different media is provided in Data S1 and Data S2.
